# Is the isolation of *S. epidermidis* from pre-operative synovial fluid culture a predictor of *S. epidermidis* prosthetic joint infection?

**DOI:** 10.1007/s10096-025-05070-3

**Published:** 2025-02-25

**Authors:** Flaminia Olearo, Carl Philipp Schoppmeier, Anna Both, Mustafa Citak, Gregor Maschkowitz, Sabine Schubert, Helmut Fickenscher, Thorsten Gehrke, Martin Aepfelbacher, Holger Rohde

**Affiliations:** 1 Institut für Medizinische Mikrobiologie, Virologie und Hygiene, Universitätsklinikum Hamburg- Eppendorf, Hamburg, Germany; 2 Abteilung für chirurgische Orthopädie, Helios ENDO-Klinik, Hamburg, Germany; 3 Medizinische Fakultät, Institut für Infektionsmedizin, Christian-Albrechts-Universität zu Kiel, Kiel, Germany

**Keywords:** Prosthetic joint infection (PJI), Synovial fluid, Microbiological diagnosis, *Staphylococcus epidermidis*, Clinical significance

## Abstract

Pathogen identification is key to the management of periprosthetic joint infection (PJI). *Staphylococcus epidermidis* is a major cause of PJI. Differentiating between invasive and contaminating *S. epidermidis* from joint specimens is challenging, and usually *S. epidermidis* is only considered a true invasive isolate when grown from two or more independent tissue samples. Thus, the detection of *S. epidermidis* in single synovial fluid (SF) samples from preoperative athrocentesis makes it difficult to draw definitive conclusions about its clinical significance, especially when grown from contamination-prone broth enrichment cultures (BEC). This study evaluates the diagnostic value of *S. epidermidis* detection in preoperative synovial cultures for the diagnosis of *S. epidermidis*-related PJI diagnosed by intraoperative tissue culture (TC). A total of 292 patients were included in the study, of whom 271 had prosthetic joint infection (PJI) according to EBJIS criteria. Preoperative synovial fluid (SF) cultures detected *Staphylococcus epidermidis* in 32.5% cases (SF-epi), other pathogens in 43.2% cases (SF-other), and were negative in 24.3%. Intraoperative tissue cultures TC identified clinically significant *S. epidermidis* in 30.1% of cases. The overall agreement between SF and tissue cultures was 66.1%, and the presence of *S. epidermidis* in SF was confirmed by TC in 70.5%. The diagnostic accuracy of SF cultures for *S. epidermidis* PJI was 83.2%, with sensitivity of 76.1% and specificity of 86.3% to detect *S. epidermidis* PJI. The positive likelihood-ration (LR+) was 5.5. When *S. epidermidis* from SF-BEC were excluded from analysis, specificity increased to 94.2%, and LR + was 7, but diagnostic sensitivity dropped to 40.5%. This study highlights the important value of using preoperative SF fluid cultures for the diagnosis of *S. epidermidis* PJI. The integration of BEC improves diagnostic accuracy and sensitivity in *S. epidermidis* PJI, and thus providing valuable information to guide clinical practice. For definitive antibiotic treatment decisions intraoperative tissue cultures remain mandatory.

## Introduction

Periprosthetic joint infections (PJI) represent a challenging medical condition associated with high morbidity, and mortality with an unadjusted 1-year and 5-year overall survival after PJI diagnosis was 88.7% and 67.2% for total hip arthroplasty and 91.7% and 71.7% for total knee arthroplasty, respectively. Furthermore, PJI incidence is expected to rise [1].

Treatment decisions, including surgical strategies and antimicrobial regimens, rely heavily on the results of pre-operative diagnostic assessments, which encompass cytology, inflammatory markers, and microbiological analyses. Among these, microbiological findings from pre-operative synovial fluid play a pivotal decisive role [2]. However, their sensitivity is regularly compromised by previous exposure to antibiotics, inadequate pre-analytical sampling and the presence of slow-growing organisms associated with biofilm-forming phenotypes [3–5]. In addition, patient samples may be contaminated with organisms derived from the skin microbiota, reducing the overall specificity of SF analysis, e.g. for *Cutibacterium spp.* or coagulase-negative staphylococci (CoNS), including *Staphylococcus epidermidis*. These low-virulence species are therefore only considered to be true invasive isolates when grown from two or more independent tissue samples [6]. In turn, the detection of *S. epidermidis* in a single synovial fluid (SF) specimen from pre-operative arthrocentesis makes it difficult to draw definitive conclusions regarding its clinical significance. Furthermore, a previous study showed a concordance of around 50% between pre-operative and intra-operative samples [7, 8], highlighting the complexity of accurately assessing the clinical relevance of *S. epidermidis* in suspected PJI cases.

The aim of this study was to evaluate the clinical significance of *S. epidermidis* isolated from pre-operative SF cultures of suspected PJI cases and to investigate the accuracy of *S. epidermidis* isolation for the diagnosis of *S. epidermidis* PJI. It was also hypothesized that the diagnostic accuracy of *S. epidermidis* isolation would depend on whether *S. epidermidis* was isolated from direct SF plating or only from broth enrichment cultures.

## Methods

### Study design and patients

We conducted a retrospective study of all adult patients who underwent surgery for revision of total hip (THA), knee (TKA), shoulder or elbow arthroplasty for suspected PJI at the Helios ENDO-Klinik, Hamburg, Germany between January 2019 and January 2021. Patients were included if pre-operative synovial fluid (SF) culture results not older than 12-month results were available, and if no pre-operative antibiotics were administered. PJI was defined according to the criteria of the European Bone and Joint Infection Society (EBJIS) [6] and PJI patients were classified according to microbiological criteria (Table 1). The study was approved by the ethics committee of the medical council Hamburg (2022-100760-BO-ff).

**Table 1:** Definitions employed to classify patient groups

Patient group	Definition
SF-epi	Patients in which *S. epidermidis* was cultured from pre-operative synovial fluid.
DC	Direct culture; patients in which *S. epidermidis* was grown from SF by direct agar plate inoculation.
BEC	Broth enrichment culture; patients in which *S. epidermidis* was only identified from SF after broth enrichment, direct cultures negative.
SF-other	Patients in which bacteria other than *S. epidermidis* were identified from pre-operative synovial fluid.
SF-negative	Patients in which synovial fluid cultures did not reveal bacterial grow.
*S. epidermidis* PJI	Patients fulfilling EBJIS AND detection of *S. epidermidis* in at least two independent intra-operative tissue samples.

### Laboratory and microbiological analysis

Synovial fluid samples were aseptically obtained prior to definitive surgery. At least one mL of the collected SF was sent for microbiological analysis. For direct culture (DC), minimum 50 µl of SF were spread onto Columbia sheep blood agar (Oxoid, Basingstoke, UK), and chocolate agar (Biomerieux, Marcy l´Étoil, France) for the detection of aerobic bacteria and yeast and on Columbia sheep blood agar for cultivation of anaerobic bacteria (all agar media from Oxoid, Basingstoke, UK). Incubation was carried out at 37 °C and 5% CO_2_ and anaerobic conditions, respectively, for up to 14 days. Plates were evaluated for growth after 24 h, 48 h, 7 days and 14 days. In addition to solid media, 9 ml of thioglycolate broth and 8 ml BHI broth (Becton Dickinson, Franklin Lakes, USA) were inoculated with up to 500 µl SF (referred to as broth enrichment culture [BEC]). All microorganisms were subjected to species identification using matrix-assisted laser desorption ionisation time-of-flight-mass spectrometry (MALDI-ToF; Microflex, Bruker Daltonics, Bremen, Germany). Antimicrobial susceptibility testing was conducted using the Vitek 2 system (Biomerieux, Marcy l´Étoil, France) or gradient diffusion (according to EUCAST protocols) when appropriate.

Isolation of *S. epidermidis* from at least two intra-operative tissue specimens from PJI cases were considered definitive *S. epidermidis* PJI. Clonal identity of *S. epidermidis* isolates from SF cultures and intra-operative tissue specimens was assumed if both isolates exhibited identical antimicrobial resistance patterns.

### Data collection

Demographic, clinical, clinical chemistry and microbiological data were collected retrospectively from medical records.

### Statistical analysis

For the descriptive analysis, normally distributed continuous variables are presented as mean ± standard deviation (SD), non-normally distributed continuous variables as median (interquartile range), and categorical variables as proportions (percentages). Differences in baseline variables between the three groups of synovial culture results (synovial fluid culture positive for *S. epidermidis*, synovial fluid culture positive for a bacterium other than *S. epidermidis*, and synovial fluid culture negative) were analysed using one-way analysis of variance (ANOVA) for continuous variables with a normal distribution (e.g. age, CRP levels and SF leukocyte count), and Kruskal-Wallis test for non-parametric continuous and ordinal variables (e.g. presence of a sinus tract and intra-operative histology positive for infection).

The value of *S. epidermidis* identification in SF to diagnose a *S. epidermidis* PJI was evaluated by assessing the sensitivity, specificity, overall accuracy and corresponding two-sided 95%-Wald confidence intervals (95% CI) compared to isolation of clinically significant *S. epidermidis* from intra-operative culture. Furthermore, to refine the diagnostic utility of *S. epidermidis* identification in SF, the Positive Likelihood Ratio (LR+) and Negative Likelihood Ratio (LR-) will also be determined. Diagnostic accuracy parameters were calculated separately for detection of *S. epidermidis* in SF cultures by (1) DC OR BEC and (2) DC only. In our analysis, a result was considered a true positive when *S. epidermidis* identified in synovial fluid (SF) matched the antimicrobial susceptibility testing (AST) profile of *S. epidermidis* from tissue culture (TC). A false positive occurred if *S. epidermidis* in SF had a differing AST profile, if SF detected *S. epidermidis* while another bacterium or no bacteria were found intra-operatively. A false negative was noted if *S. epidermidis* appeared only in the intra-operative culture but not in SF. A true negative was recorded when a different bacterium was isolated from both SF and TC, or both were sterile, with no agreement sought on the identify of any other isolated bacteria.

The agreement between *S. epidermidis* isolation from SF and intra-operative tissue specimens was analysed by calculating Cohen´s kappa coefficient κ, and interpreted according to published criteria (κ ≤ 0, no agreement; κ = 0.01–0.20, none to low agreement; κ = 0.21–0.40, fair agreement; κ = 0.41–0.60, moderate agreement; κ = 0.61–0.80, substantial agreement; κ = 0.81-1.00, almost perfect agreement [9]). Culture results were considered concordant if SF and intra-operative tissue samples were identified as clonally identical *S. epidermidis* in both materials (at least two intra-operative samples tested positive for *S. epidermidis*). All *S. epidermidis* isolates without antimicrobial susceptibility testing were excluded from the study.

## Results

Overall, 292 patients were included in this study. Table 2 shows the clinical characteristics of patients by results of the pre-operative synovial fluid culture (presence of *S. epidermidis* [SF-epi], other pathogens [SF-other] and culture negative [SF-negative]). Definitive PJI was retained in 271/292 (92.8%) patients, 8 (2.7%) possibly infected, 12 (4.1%) not infected and 1 (0.3%) inconclusive according to EBJIS criteria.

**Table 2:** Clinical characteristics by type of SF culture results: *S. epidermidis*, culture-negative, and culture-positive (other than *S. epidermidis*)

Variables	SF-epi patients (*N* = 95)	*S*ynovial fluid culture negative (*N* = 71)	Positive synovial fluid culture with a pathogen other than *S. epidermis* (*N* = 126)	Comparison between the three groups, *p*-value
Etiology, N (%)	*S. epidermidis*	Not applicable	*Cutibacterium spp.*, 35 (27.7) Coagulase negative staphylococci, 32 (25.4) *Streptococcus spp*., 24 (19) *S. aureus*, 14 (11.1) *Enterococcus spp*., 5 (4) *E. coli*, 5 (4) *P. mirabilis*, 4 (3.2) *K. pneumoniae*, 1 (0.8) *P. aeruginosa*, 1(0.8) Other, 5 (4)	
Gender female, N (%)	44 (46.3)	31 (43.6)	43 (34.1)	0.148
Age years, mean (SD)	66.5 (11.7)	65 (10.7)	68.2 (10.79)	0.14
Type of articulation, N (%):				0.524
Hip	61 (64.2)	43 (60.6)	80 (63.5)	
Knee	32 (33.7)	24 (33.8)	40 (31.7)	
Shoulder	1 (1.1)	4 (5.6)	6 (4.8)	
Elbow	1 (1.1)			
Clinical manifestation of PJI, Acute, N (%) *2 missing values*	17 (17.9)	40 (57.1)	74 (59.2)	**< 0.001**
Presence of sinus tract, N (%)	23 (24.2)	17 (23.9)	28 (22.2)	0.95
Intra-operative histology positive for infection, N (%) *21 missing values*	69 (77.5)	47 (72.3)	89 (76.1)	0.788
EBJIS criteria (%):				**0.026**
- Infected	87 (91.6)	62 (87.3)	122 (96.8)	
- Possibly infected	1 (1)	5 (7)	2 (1.6)	
- Not infected	7 (7.2)	4 (5.6)	1 (0.8)	
- Inconclusive	0	0	1 (0.8)	
Surgical procedures:				
DAIR	2 (2.1)	21 (29.6)	15 (11.9)	
One-stage Exchange	87 (91.6)	35 (49.3)	103 (81.7)	
Two-stage Exchange	6 (6.3)	15 (21.1)	8 (6.4)	
Perioperative markers of synovial fluid:				
- CRP, mg/L, mean (SD), serum *24 missing values*	32.9 (32.1)	28.1 (43)	62.9 (78)	**< 0.001**
- α-defensin positive, N (%) *23 missing values*	26 (31.3)	6 (9)	43 (35.8)	**< 0.001**
- LE Test, N (%)				
*86 missing values*				
0	7 (10.3)	15 (33.3)	11 (11.8)	
+	2 (2.9)	2 (4.4)	2 (2.1)	**0.036**
++	7 (10)	4 (8.9)	8(8.6)	
+++	52 (76.5)	24 (53.3)	72 (77.4)	
- PMN, %				
<=65	1 (1.6)	13 (27.7)	6 (7.8)	**< 0.001**
65 < PMN%<80	4 (6.5)	15 (31.9)	6 (7.8)	
>=80	57 (91.9)	19 (40.4)	65 (84.4)	
*108 missing values*				
- Leucocytes count, cells/µL:				
<=1500	1 (1.7)	12 (22.2)	8 (9.9)	**0.001**
1500 < Leucocytes < 3000	5 (6.7)	3 (5.6)		
> 3000	55 (91.7)	39 (72.2)	73 (90.1)	
*107 missing values*				
*S. epidermidis* PJI confirmed by intra-operative culture, N (%) *N* = 88	75 (83.3)	12 (16.9)	*C. acnes* (pre-operative) 1 (0.8)	
PJI with an intra-operative culture positive for a pathogen other than *S. epidermidis*, *N* = 149	*E. coli*, 1 *K. pneumoniae*, 1 *F. magna*, 1 *C. acnes*, 1 *S. capitis* 1 *S. dysgalactiae* 1	*S. aureus*, 7 *C. acnes*, 6 Gram-negative bacilli, 5 Coagulase-negative staphylococci, 10 *Streptococcus spp*., 3 Other, 7	*Pre-operative versus intra-operative discordant culture results*: - *C. avidum* (*S. haemolyticus* in SF), 1 - *S. caprae* (*P. mirabilis* in SF), 1 - *K. aerogenosa* (*S.capitis* in SF),1 - *E. faecalis* (*S. hominis* in SF),1 - *S. agalatiae* (*E. faecalis* in SF), 1	

### Detection of S. epidermidis in SF

*S. epidermidis* was identified in pre-operative SF cultures in 95/292 cases (32.5%). In 42/95 (44.2%) cases, *S. epidermidis* was isolated from direct cultures (DC), while 53/95 (55.8%) were from broth enrichment cultures (BEC) only. 63/95 (66.3%) *S. epidermidis* isolates were methicillin resistant (29/42 [69.1%] and 34/53 [64.2%] of DC and BEC isolates, respectively).

SF cultures grew pathogens other than *S. epidermidis* in 126 (43.2%) of cases (referred to as SF-other), and 71 cases (24.3%) had a negative synovial culture result (referred to as SF-negative). The most prevalent pathogen in the SF-other group was *Cutibacterium spp.*, identified in 35 cases (27.7%), followed by Coagulase-negative staphylococci other than *S. epidermidis* (25.4%) and *Streptococcus spp.* (19%).

There were no statistically significant differences in the distribution of gender, age, joint involved, intra-operative histology positive for infection and presence of sinus tract among the SF-epi, SF-other and SF-negative groups. SF markers indicative for inflammation were lower in the SF-negative group (Table 2). Intriguingly, the percentage of patients from the SF-negative group with a definitive PJI diagnosis was statistically significant lower compared to the SF-epi and the SF-other group (87.3%, 91.6%, 96.8%, respectively). 12/294 patients did not met criteria for PJI according EBJIS criteria, respectively (SF-epi, 7 [7.2%]; SF-other 1 [0.8%]; SF-negative, 4 [5.6%]). Debridement, antibiotic and implant retention (DAIR) was performed in 38/292 (13%), one-stage exchange in 225/292 (77.1%), and two-stage exchange in 29/292 (9.9%).

### Detection of clinically significant S. epidermidis from intra-operative tissue specimens

The median number of intra-operative tissue samples collected was 8 (IQR 7–10). Clinically significant *S. epidermidis* (i.e., recovery of *S. epidermidis* from two independent tissue specimens) were identified in 88/292 (30.1%) patients (Table 2), proving *S. epidermidis* PJI (referred to as TC-epi; Table 1). In 149 patients tissue specimens grew pathogens other than *S. epidermidis* (referred to as TC-other), and in 55 cases tissue culture revealed no bacterial growth (referred to as TC-negative). Among the *S. epidermidis* isolated from intra-operative tissue specimens, 61/88 (69.3%), were methicillin resistant.

### Comparison of SF-epi with culture findings from tissue specimens

Overall comparison of microbiological results from SF cultures with results from intra-operative specimens found an overall agreement between pre-operative SF cultures and intra-operative tissue cultures in 193/292 (66.1%). Detailed analysis focussing on SF-epi cases (*n* = 95) and taking AST profiles into account to rate clonal identity of *S. epidermidis* isolates showed that in 67/95 (70.5%) (DC, *n* = 34; BEC, *n* = 33), presence of *S. epidermidis* was confirmed by intra-operative cultures. In 8/95 (8.4%), *S. epidermidis* from SF and TC exhibited differences in AST profiles were thus deemed unrelated. In 6/95 (6.3%) SF-epi patients, intra-operative tissue samples grew other organisms than *S. epidermidis* (DC, *n* = 1; BEC, *n* = 5), and in 14 SF-epi patients, tissue cultures remained negative. Vice versa, SF did not grow *S. epidermidis* in 12/88 cases with clinically significant *S. epidermidis* (Table 3).

**Table 3:** Comparative representation of microbiological findings from SF and tissue cultures ^a^

	SF epi (*n* = 95)	SF other (*n* = 126)	SF negative (*n* = 71)
TC epi (*n* = 88)	75^b^	1	12
TC other (*n* = 149)	6	105	38
TC negative (*n* = 55)	14	20	21

^a^ Based on species identification only

^b^ In 8 / 75 cases, AST profiles differed between *S. epidermidis* from SF or TC cultures

The accuracy of SF cultures to diagnose a *S. epidermidis* PJI was 83.2% (95%CI: 78.4–87.3) (Table 4). The sensitivity and specificity were 76.1% (95%CI: 65.9–84.6) and 86.3% (95%CI: 80.8–90.7), respectively. Considering only SF-epi cases with positive DC, the specificity increased up to 94.2% (95%CI: 90.1–97). However, the sensitivity dropped to 40.5% (95%CI: 29.9–51.7), and the accuracy decreased to 78.8% (95%CI: 73.6–83.3) (Table 4). Interestingly, when considering only *S. epidermidis* isolated from DC, the positive likelihood ration (LR+) was 7, indicating that *S. epidermidis* isolated in SF is seven time more likely compared to baseline for this study population. When DC and BEC are considered, the LR + dropped to 5.5, still indicating a moderate to strong diagnostic usefulness to predict a *S. epidermidis* PJI. Cohen´s Kappa analysis showed a strong agreement between SF cultures and intra-operative cultures if DC and BEC were combined (κ = 0.67, percent agreement 83.2%), while when just DC were considered, it was fair (κ = 0.4, percent agreement 78.8%).

**Table 4:** Diagnostic accuracy of *S. epidermidis* positive synovial fluid culture in adult patients with *S. Epidermidis* PJI (based on the phenotype)

Type of synovial fluid culture	S. epidermidis PJI						
	Accuracy % (95% CI)	Sensitivity % (95% CI)	Specificity % (95% CI)	PPV % (95% CI)	NPV % (95% CI)	Positive Likelihood Ratio	Negative Likelihood Ratio
*S. epidermidis* isolated by DC OR BEC % (CI 95%) *N* = 292 *True positives: 67* *False positives: 28* *True negatives: 176* *False negatives: 21*	83.2 (78.4–87.3)	76.1 (65.9–84.6)	86.3 (80.8–90.7)	70.5 (62.5–77.5)	89.3(85.2–92.4)	5.5 (3.9-8)	0.3 (0.2–0.4)
*S. epidermidis* isolated DC % (CI 95%) *N* = 292 *True positives: 34* *False positives: 8* *True negatives: 196* *False negatives: 54*	78.8 (73.6–83.3)	40.5 (29.9–51.7)	94.2 (90.1–97)	73.9 (60.7–83.9)	79.7 (76.6–82.4)	7 (3.8–12.9)	0.6 (0.5–0.8)

## Discussion

Pathogen detection is a fundamental component of the diagnosis of PJI: it serves as a key infection-defining criterion, provides critical information to guide targeted antimicrobial therapy, and facilitates decisions about the most appropriate surgical treatment strategy. While for definitive microbiological diagnosis, at least for low-virulent organisms, invasive measures (i.e. arthrotomy) is necessary, SF is a relatively easily accessible specimen for diagnostic evaluation in PJI. In fact, pre-operative aspiration of synovial fluid is a recommended by EBJIS. According to the EBJIS criteria for diagnosing a PJI [6], a positive synovial fluid culture in combination with at least one additional diagnostic finding is a classifier for a likely PJI. However, due to the significant possibility of contamination during sample collection and processing, it is not without risk to rely on a single positive SF puncture to detect the PJI causing organism. This is particularly relevant for the identification of *S. epidermidis*, the most typical cause of specimen contamination in clinical microbiology [10]. Given the significant implications for patient care, we therefore evaluated the diagnostic accuracy of *S. epidermidis* detection in SF cultures to predict a PJI caused by *S. epidermidis*. Enrichment techniques are used to increase diagnostic sensitivity, however, in cases where only BEC yields growth of *S. epidermidis*, the clinical significance of the isolate is often in doubt. For this reason, our analysis distinguished between cases in which *S. epidermidis* was detected by solid agar cultures or by broth enrichment cultures.

Overall, taking into account all culture-positive and -negative cases, our study showed an agreement of microbiological findings from SF-C and TC in 66%. This is consistent with published studies reporting an overall agreement of SF-C and TC in 52–79% [11–14]. For definitive *S. epidermidis* PJI cases, we found an overall agreement between SF-C and TC of 70.5%. Intriguingly, evidence from the literature for the agreement of *S. epidermidis* detection in SF-C and TC is scarce, and the agreement ranges from 35 to 95% [7, 15]. There are many possible reasons for the considerable differences in the reported concordance of SF-C and TC in general, and in particular for CoNS and *S. epidermidis*. Both the sensitivity and specificity of culture analysis are determined by aspects of sample transport (storage time, transport time), specimen processing in the laboratory (e.g. sample processing [e.g. tissue homogenisation, SF centrifugation]) and culture conditions (e.g. sample volume used for growth media inoculation, type of culture media used, duration of incubation) [5, 16]. For the daily praxis, as these aspects may vary significantly between centres, it is thus important to evaluate local diagnostic workflows and procedures to gain information on how to judge positive and negative SF or TC cultures in general.

A major finding of our study is that the specificity, +LR and accuracy of detection of *S. epidermidis* in SF-C are clearly dependent on whether the organisms can be detected on solid agar plates or only after broth enrichment. Obviously, during clinical workup a more detailed assessment of microbiological results is necessary for correct interpretation of clinical significance: i.e. the high specificity (94.2%) of *S. epidermidis* SF-DC to detect proven *S. epidermidis* PJI is a strong indication of clinical significance. On the other hand, the lower specificity (76.1%) shows that while BEC are necessary to achieve an acceptable sensitivity and accuracy, the clinical significance of *S. epidermidis* from SF-BEC is questionable. Thus, in those cases clinical findings and SF parameters (i.e. leukocyte esterase, leukocyte count, α-defensin) must be considered to allow for correct interpretation of clinical findings.

As a consequence of our findings, it appears worthwhile to update EBJIS criteria for likely PJI by revisiting the SF culture criterion through integration of a quantitative dimension to microbiological findings. This should be considered at least for low virulent organisms from skin microbiota, and may help to increase accuracy of EBJIS PJI definition. In more general terms, our findings for *S. epidermidis* cultures indicate the urgent need to establish a rigorous, unambiguous microbiological definition of a positive culture in PJI diagnostics.

This study has several limitations. Not all patients undergoing initial SF analysis for PJI diagnostics were included, but only those who underwent subsequent surgery. Moreover, the relative distribution of pathogens isolated from PJI differed from other reported series [17], with an over-representation of infections related to low-virulent pathogens. With regard to microbiological analysis, it needs to be stressed that we relied on phenotypic criteria to assess the clonal identity of *S. epidermidis* from SF or TC. Recent studies indicate that due to ongoing genetic adaption during infection, phenotypic resistance profiles may change significantly over the course of an infection, questioning the precision of that criterium ([18, 19]. Thus, for unambiguous determination of clonal relationship of *S. epidermidis* from SF cultures and TC appears, in future studies whole genome sequencing is mandatory. Moreover, we applied strict criteria to define *S. epidermidis* PJI (i.e. detection of *S. epidermidis* from two independent biopsies), and it cannot be ruled out that this led to an underestimation of the true prevalence of *S. epidermidis* PJI in the study population.

In conclusion, our data support the concept that pre-operative SF cultures are useful to identify causative pathogens in a significant number of PJI cases. Specifically, detection of *S. epidermidis* in direct cultures is indicative of a *S. epidermidis* PJI, but diagnostic sensitivity is low. To achieve a clinically useful diagnostic sensitivity and accuracy, at least for *S. epidermidis*, a combination of DC and BEC is necessary. However, related to the low specificity associated with this microbiological approach, a comprehensive patient assessment including clinical and SF parameters is mandatory to judge for clinical significance. Furthermore, at least for *S. epidermidis* it is questionable to what extent decisions on antimicrobial treatment strategies can be based on preoperative SF cultures.

## Data Availability

No datasets were generated or analysed during the current study.
